# Progression of Nonmotor Symptoms in Parkinson's Disease by Sex and Motor Laterality

**DOI:** 10.1155/2021/8898887

**Published:** 2021-04-01

**Authors:** Zimple Kurlawala, Paul H. Shadowen, Joseph D. McMillan, Levi J Beverly, Robert P. Friedland

**Affiliations:** ^1^Diabetes and Obesity Center, University of Louisville, Louisville, KY 40292, USA; ^2^School of Medicine, University of Louisville, Louisville, KY 40292, USA; ^3^Department of Molecular Medicine, University of South Florida, Tampa, FL, USA; ^4^Department of Neurology, University of Louisville, Louisville, KY 40292, USA

## Abstract

Nonmotor symptoms (NMS) in Parkinson's disease (PD) can start up to a decade before motor manifestations and strongly correlate with the quality of life. Understanding patterns of NMS can provide clues to the incipient site of PD pathology. Our goal was to systematically characterize the progression of NMS in PD (*n* = 489), compared to healthy controls, HC (*n* = 241), based on the sex of the subjects and laterality of motor symptom onset. Additionally, NMS experienced at the onset of PD were also compared to subjects with scans without dopaminergic deficit, SWEDD (*n* = 81). The Parkinson's Progression Markers Initiative (PPMI) database was utilized to analyze several NMS scales. NMS experienced by PD and SWEDD cohorts were significantly higher than HC and both sex and laterality influenced several NMS scales at the onset of motor symptoms. *Sex Differences*. PD males experienced significant worsening of sexual, urinary, sleep, and cognitive functions compared to PD females. PD females reported significantly increased thermoregulatory dysfunction and anxious mood over 7 years and significantly more constipation during the first 4 years after PD onset. *Laterality Differences*. At onset, PD subjects with right-sided motor predominance reported significantly higher autonomic dysfunction. Subjects with left-sided motor predominance experienced significantly more anxious mood at onset which continued as Parkinson's progressed. In conclusion, males experienced increased NMS burden in Parkinson's disease. Laterality of motor symptoms did not significantly influence NMS progression, except anxious mood. We analyzed NMS in a large cohort of PD patients, and these data are valuable to improve PD patients' quality of life by therapeutically alleviating nonmotor symptoms.

## 1. Introduction

Parkinson's disease (PD) is a neurodegenerative disorder clinically associated with three cardinal symptoms–bradykinesia, rigidity, and rest tremor. Although PD is known for its hallmark motor symptoms, PD has both, motor and nonmotor manifestations. Some of the important nonmotor symptoms (NMS) include dysfunction associated with cognition, sleep, mood, vision, sex, impulse control, sense of smell, and cardiovascular, gastrointestinal, and genitourinary systems [1]. These prodromal NMS can start up to a decade before motor manifestations and exhibit a stronger correlation with the quality of life in PD [2]. NMS often do not respond to standard dopaminergic therapy and may actually be worsened by this treatment [3]. Understanding the characteristics and patterns of NMS is valuable from a therapeutic point of view, and it may also provide clues to the incipient site of PD pathology.

In this pursuit, we aimed to characterize NMS in Parkinson's disease. Asymmetry of motor symptoms is a perennial feature of PD and is observed in 85% of PD patients at onset [4]. Asymmetry has become a part of the Movement Disorder Society's Clinical Diagnostic Criteria for the disease [5]. Previous attempts have been made to study NMS based on motor laterality [6–10], sex [11–19], and handedness [4, 20–23]. However, most of these studies evaluated NMS only at onset and sample sizes were not large enough resulting in conflicting conclusions.

In this manuscript, we conducted a systematic review of NMS utilizing a large cohort from Parkinson's Progression Markers Initiative (PPMI) database at onset and progression over 7 years in PD (*n* = 489) and healthy controls (HC) (*n* = 241). We also included NMS data at onset from subjects with scans without dopaminergic deficit (SWEDD) (*n* = 81). SWEDD subjects experience PD-like symptoms, both motor and nonmotor, but do not show a dopaminergic deficit in their DaTSCAN as one would expect in PD.

## 2. Materials and Methods

### 2.1. Study Population

We utilized Parkinson's Progression Markers Initiative (PPMI) cohort, an ongoing observational clinical study, enrolling patients since 2010 at multiple international sites. We accessed the database on June 12^th^, 2018. We analyzed 241 healthy controls, 489 Parkinson's disease subjects, and 81 subjects categorized as SWEDD (scans without evidence of dopaminergic deficit). PPMI data collection was approved by the Institutional Review Boards at all clinical sites.

### 2.2. Inclusion and Exclusion Criteria

Participants were divided into cohorts including healthy controls (HC), *de novo* PD subjects (PD), and subjects without evidence of dopaminergic deficit (SWEDD), among other categories. Individuals in the HC cohort did not have an active clinically significant neurological disorder, a first degree relative with idiopathic PD, or a Montreal Cognitive Assessment (MoCA) score ≤ 26. Individuals in the PD cohort had at least two of the cardinal signs of PD (rest tremor, bradykinesia, or rigidity) or either asymmetric rest tremor or asymmetric rigidity. All diagnoses were supported by neuroimaging results consistent with a dopamine transporter deficit. Participants had also been diagnosed with PD for 2 years or less at the time of screening, were not taking PD medications, and were not expected to need PD medications within the first 6 months of the study. As the disease progressed, most subjects started Levodopa or a dopamine agonist. Subjects who had been diagnosed with PD before the age of 30 were excluded. Individuals in the SWEDD cohort had the same inclusion criteria as the PD cohort; the only difference was that they also had a screening dopamine transporter SPECT scan that did not show any evidence of dopamine transporter deficit. Subjects in all cohorts were 30 years of age or older at the time of screening.

Onset analysis: most of the data analyzed for onset analysis came from the baseline visit. The screening visit occurred 45 days before the baseline visit. However, MoCA and Geriatric Depression Scale were measured during the screening visit, but not again at the onset visit. We analyzed what was collected at the screening visit for those two measures for Table 1.

### 2.3. Measures

PPMI cataloged an extensive array of motor and nonmotor symptoms experienced by subjects. We appraised nonmotor symptoms via MDS-UPDRS, the University of Pennsylvania Smell Identification Test (UPSIT), Montreal Cognitive Assessment (MoCA), Geriatric Depression Scale (GDS), REM Sleep Behavior Disorder Screening Questionnaire (RBDSQ), Questionnaire for Impulsive-Compulsive Disorders (QUIP-S), and Scales for Outcomes in Parkinson's Disease (SCOPA-AUT). Demographic data and features of PD onset were available in the medical history information provided by each participant. We specifically reviewed all measures as they were recorded during subjects' initial study visits (screening or onset). Where sufficient data was available, we analyzed the progression of select nonmotor symptom from the initial visit to the fourteenth visit (7 years from onset).

NMS scales (range of scores and subscales displayed in figures).

(1) MDS-UPDRS1 (0–52) (Movement Disorder Unified Parkinson's Disease Rating Scale Part I):
  - Anxious mood (0–4)
  - Pain and other sensations (0–4)
  - Constipation problems (0–4)
(2) SCOPA-AUT (0–69) (Scales for Outcomes in Parkinson's Disease: Autonomic Version):
  - Cardiovascular subscore (0–9)
  - Gastrointestinal subscore (0–21)
  - Pupillomotor (0–3)
  - Sexual (0–13)
  - Thermoregulatory (0–12)
  - Urinary subscore (0–18)
(3) UPSIT (0–40) (University of Pennsylvania Smell Identification Test)
(4) GDS (0–15) (Geriatric Depression Scale)
(5) MoCA (0–30) (Montreal Cognitive Assessment):
  - MoCA Recall (0–5)
(6) RBDSQ (0–10) (REM Sleep Behavior Disorder Screening Questionnaire)
(7) QUIP (0–13) (Questionnaire for Impulsive-Compulsive Disorders in Parkinson's Disease)

### 2.4. Statistical Analysis

We evaluated the association of laterality of motor symptoms at PD onset with nonmotor symptoms. Scale variables were first examined using the Shapiro–Wilk test for normality. Associations involving all scale variables were examined using the nonparametric independent-samples Mann–Whitney *U* test. Overall differences at onset between the HC, PD, and SWEDD cohorts were evaluated using the nonparametric independent-samples Kruskal–Wallis test; pairwise differences were evaluated using the nonparametric independent-samples Mann–Whitney *U* test with Bonferroni correction for multiple comparisons. Changes in the mean prevalence of symptoms over time were compared using the Shapiro–Wilk test for normality followed by two-way ANOVA. For all tests, statistical significance was defined as *p* < 0.05, except where indicated. Statistical analyses were conducted using IBM SPSS Statistics (version 25), GraphPad Prism (version 7), and Microsoft Office Excel (version 16.13.1).

## 3. Results

### 3.1. Subject Characteristics

Our analyses included a total of 811 subjects in three cohorts: 241 healthy controls (HC), 489 Parkinson's disease subjects (PD), and 81 SWEDD. For a complete list of inclusion criteria, please refer to Materials and Methods.

Onset demographics and disease characteristics for each cohort are provided in Table 1. Study participants were mostly Caucasian (91%), males (64%), and right-handed (>80%), and the mean age of onset of motor symptoms was 59.2 years. Most PD and SWEDD subjects were in Hoehn and Yahr stage I or II of disease progression at the time of enrollment. Within the PD cohort, motor symptom predominance was reported as right in 55%, left in 42%, and bilateral in 3%. Within the SWEDD cohort, motor symptom predominance was reported as right in 65%, left in 27%, and bilateral in 7%. For data analyses, we excluded cases with symmetrical motor predominance.

### 3.2. Influence of Sex on NMS: Onset Analysis

**Figure 1:**
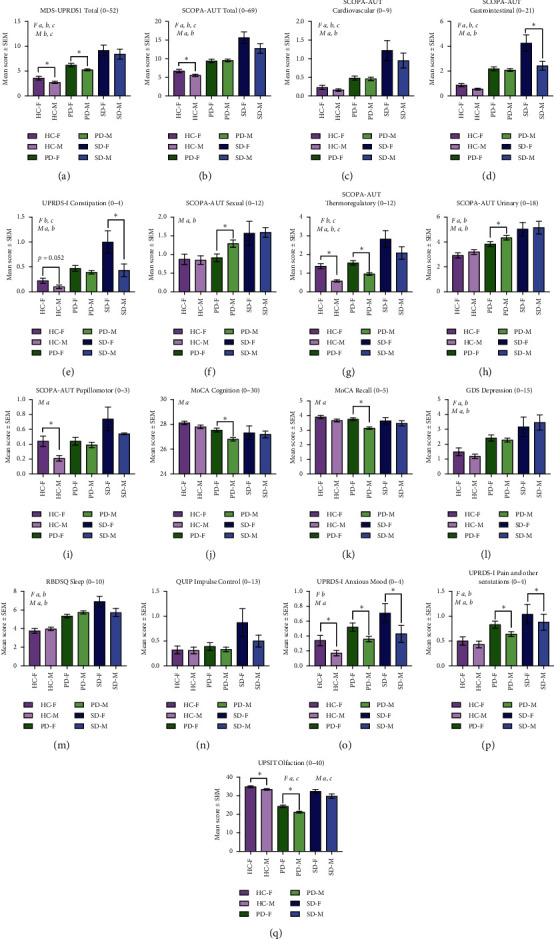
Prevalence of NMS at onset, categorized by sex. NMS mean scores were categorized by sex of participant (female (F) or male (M)) for Parkinson's disease (PD), SWEDD (SD), and healthy controls (HC). NMS scales analyzed are displayed A through Q indicating the score range in the title. Higher number indicates more dysfunction except for MoCA Cognition (J) and USPIT Olfaction (O). Pairwise differences within the cohort (females vs. males) were evaluated with independent-samples Mann–Whitney *U* test with Bonferroni correction for multiple comparisons. Differences between cohorts (HC vs. PD vs. SWEDD) for each sex were evaluated using the independent-samples Kruskal–Wallis test. Statistical differences within cohorts (females (v) males) are indicated by an asterisk  ^*∗*^. Post hoc analyses signifying differences between the HC, PD, and SWEDD cohorts are indicated for females (F) and males (M) as *a* = HC versus PD, *b* = HC versus SWEDD, and *c* = PD versus SWEDD. *p* < 0.05.

We compared mean values for NMS for the 3 cohorts categorized by sex of subject (female or male) (Figure 1).

Within the PD cohort, females reported significantly higher mean scores for overall autonomic dysfunction (Figure 1(a)), thermoregulatory dysfunction (Figure 1(g)), anxious mood (Figure 1(o)), and pain and other sensations (Figure 1(p)). Males reported a significantly higher degree of sexual dysfunction (Figure 1(f)), urinary dysfunction (Figure 1(h)), cognitive dysfunction (Figures 1(j) and 1(k)), and hyposmia (Figure 1(q)).

Within the SWEDD cohort, females reported significantly higher mean scores for gastrointestinal dysfunction (Figures 1(d) and 1(e)), anxious mood (Figure 1(o)), and pain and other sensations (Figure 1(p)).

### 3.3. Influence of Sex on NMS: Progression Analysis

**Figure 2:**
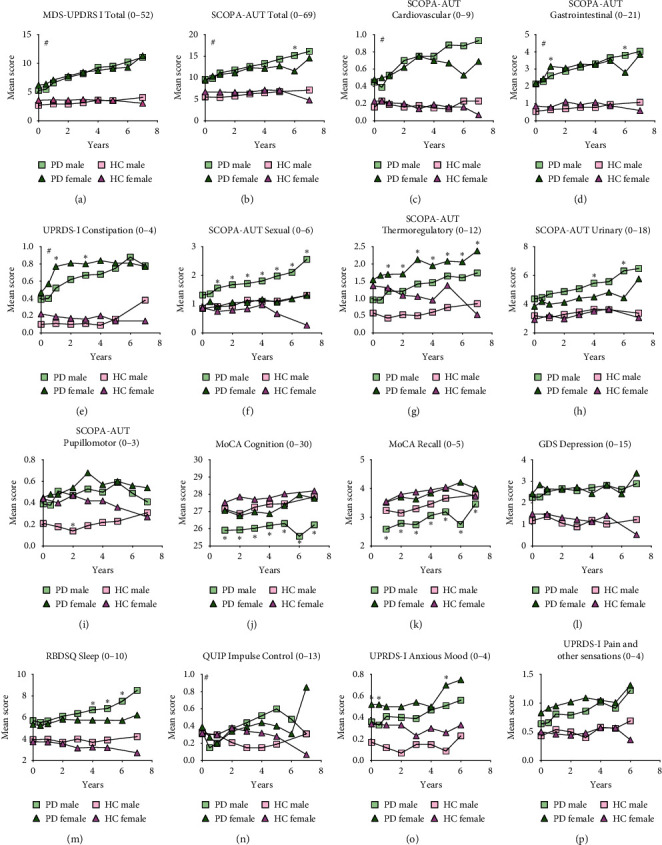
Progression of NMS in PD cohort categorized by sex. NMS mean scores were categorized by sex of patient (female (F) or male (M)) for Parkinson's disease (PD) and healthy controls (HC). NMS scales analyzed are displayed A through P indicating the score range in the title of individual graphs. Higher number indicates more dysfunction except for MoCA Cognition (J). Differences in mean scores within PD and HC cohorts (females vs. males) were evaluated using two-way ANOVA with multiple comparisons for each time point. Statistically significant differences within cohorts (F vs. M) are indicated by an asterisk ^*∗*^. Statistically significant ANOVA interaction of time and cohort on NMS scale are indicated by #*p* < 0.05.

We analyzed mean values of NMS scores for HC and PD cohorts for up to 7 years (Figure 2) but not for SWEDD cohorts due to lack of sufficient data (SWEDD patients completed only 2 follow-up visits, i.e., 2 years at the time of analyses).

Within the PD cohort, females experienced more constipation (Figure 2(e)), thermoregulatory dysfunction (Figure 2(g)), and anxious mood (Figure 2(o)). Males experienced more sexual (Figure 2(f)), urinary (Figure 2(h)), cognitive (Figures 2(j) and 2(k)), and sleep dysfunction over 7 years.

### 3.4. Influence of Laterality on NMS: Onset Analysis

**Figure 3:**
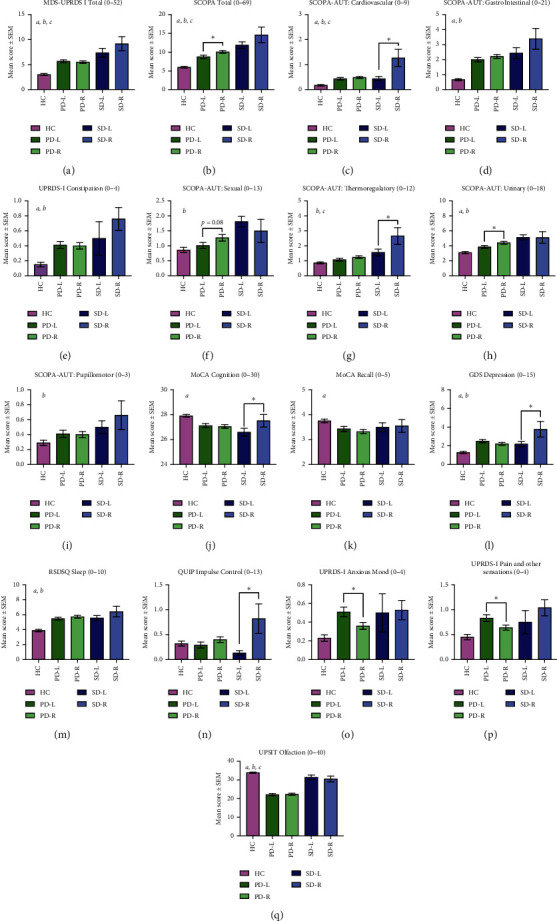
Prevalence of NMS at onset, categorized by laterality of motor symptoms. NMS mean scores were categorized by laterality of motor symptom onset (left (L) or right (R)) for Parkinson's disease (PD) and SWEDD (SD) along with healthy controls (HC). NMS analyzed are displayed A through Q indicating the score range in the title. Higher number indicates more dysfunction except for MoCA Cognition (J) and USPIT Olfaction (O). Pairwise differences within cohorts (left vs. right) were examined using the nonparametric independent-samples Mann–Whitney *U* test. Overall differences between cohorts (HC vs. PD vs. SWEDD) were evaluated using the nonparametric independent-samples Kruskal–Wallis test. Post hoc analyses signifying differences between the HC, PD, and SWEDD cohorts are indicated as *a* = HC versus PD, *b* = HC versus SWEDD, and *c* = PD versus SWEDD. *p* < 0.05.

We compared mean values for NMS for the 3 cohorts categorized by laterality of motor symptoms at onset (left or right) (Figure 3). As expected, post hoc analyses demonstrated that PD and SWEDD cohorts experienced significantly higher nonmotor symptoms than healthy controls. The most unique NMS within the PD cohort was a significant degree of hyposmia (Figure 1(q)), clearly distinguishing PD from the HC and SWEDD cohorts.

Within the PD cohort, subjects with right-sided motor predominance had significantly higher mean scores for overall autonomic (Figure 1(b)), sexual (Figure 1(f)), and urinary dysfunction (Figure 1(h)). Parkinson's subjects with left-sided motor predominance had significantly higher mean scores for anxious mood (Figure 1(o)) and pain and other sensations (Figure 1(p)).

Within the SWEDD cohort, subjects with right-sided motor predominance experienced the most severe NMS. They had higher mean scores for cardiovascular dysfunction (Figure 1(c)), thermoregulatory dysfunction (Figure 1(g)), depressed mood (Figure 1(l)), and impulse control dysfunction (Figure 1(n)). Subjects with left-sided motor predominance experienced more cognitive decline (Figure 1(j)).

### 3.5. Influence of Laterality on NMS: Progression Analysis

**Figure 4:**
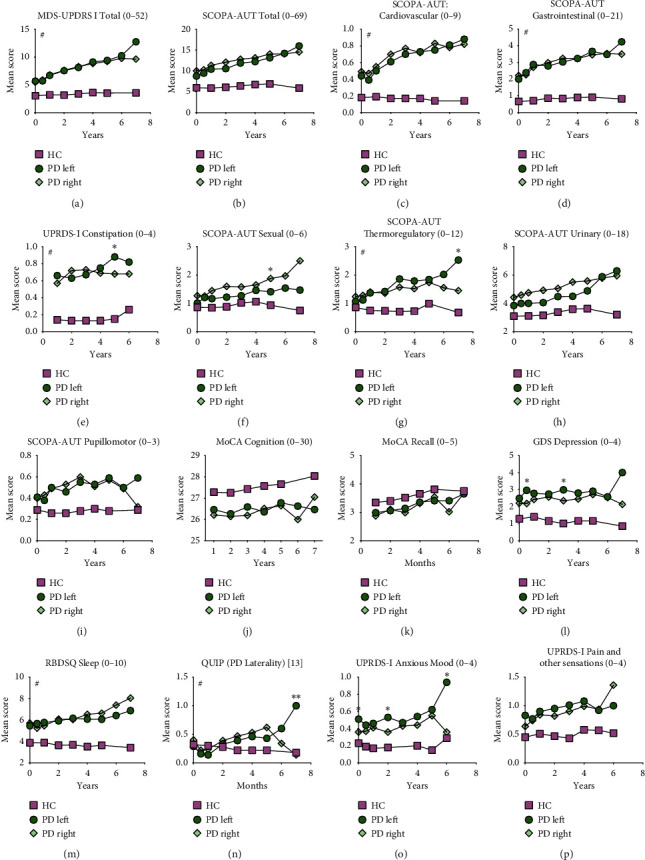
Progression of NMS in PD cohort categorized by laterality of motor symptoms at onset. NMS mean scores were categorized by laterality of motor symptom onset (left or right) for Parkinson's disease (PD) and healthy controls (HC) and mean scores for each scale were graphed for follow-up visits up to 7 years. NMS scales analyzed are displayed A through P indicating the score range in the title of individual graphs. Higher number indicates more dysfunction except for MoCA Cognition (J). Differences in mean scores within PD cohort (left vs. right) were evaluated using two-way ANOVA with multiple comparisons for each time point. Statistically significant differences between PD Left and PD Right are indicated by an asterisk ^*∗*^. Statistically significant ANOVA interaction of time and cohort on NMS scale are indicated by #. *p* < 0.05.

We analyzed mean values of NMS scores for HC and PD cohorts for up to 7 years (Figure 4) but not for the SWEDD cohorts due to lack of sufficient data (SWEDD patients completed only 2 follow-up visits, i.e., 2 years at the time of analyses). Regardless of the side that motor symptoms first appeared on, all patients experienced NMS over the course of disease development.

PD subjects with right-sided motor predominance at onset experienced progressively worse sexual symptoms (Figure 4(f)) but these data are most likely skewed towards males who experienced more sexual and urinary dysfunction compared to females (64.2% of the sample were males). PD subjects with left-sided motor predominance continued to experience increased anxious mood from onset into disease progression. For other NMS scales, this group did not exhibit clear gradual trends of worsening symptoms. However, at several indicated time points in the figure, subjects with left-sided motor predominance experienced more constipation (Figure 4(e)), thermoregulatory dysfunction (Figure 4(g)), depressed mood (Figure 4(l)), and impulse control dysfunction (Figure 4(n)) which may not be clinically significant.

## 4. Discussion

Parkinson's disease patients display a variety of motor and nonmotor symptoms. The cause of PD is attributed to genetic causes in less than 10 percent of cases; therefore, most cases are sporadic, and the exact cause is not known. Some nonmotor symptoms, such as constipation and hyposmia, can precede the hallmark PD motor signs by almost a decade. In pursuit of discovering a common pattern and identifying characteristically unique subsets of patients, we analyzed a large dataset obtained from the PPMI database and examined the prevalence and progression of NMS categorized by sex of subjects and laterality of motor symptoms in Parkinson's disease. We discovered that PD patients reported a higher degree of NMS abnormalities on almost all nonmotor scales measured, and sex of the patient had a strong influence on NMS progression.

Asymmetry of motor symptoms in Parkinson's disease is a result of asymmetric degeneration of the nigrostriatal pathway which receives motor input from the striatum in the basal ganglia. This group of nuclei are centrally situated at the base of the forebrain and have robust connections with the sensorimotor cortex, thalamus, substantia nigra, amygdala, and pallidum. Degeneration of the substantia nigra leads to impairment in the functional connectivity between these regions which may be the cause of several nonmotor symptoms in Parkinson's disease. Additionally, the involvement of the hypothalamus (control of sleep and circadian rhythm) may be central to the causation of NMS in PD. Sleep influences the circadian rhythm, which is responsible for regulating several autonomic parameters such as body temperature, cortisol secretion, melatonin secretion, blood pressure, and gene expression, which can directly or indirectly lead to autonomic and multisystem dysfunction seen in PD [24–29].

### 4.1. PD Cohort

The NMS that distinguished this cohort most significantly was hyposmia. As Parkinson's progressed, most subjects demonstrated increasing mean values on several NMS scales. Sex of the subjects had a substantial impact on the progression of nonmotor symptoms. At onset, females across all three cohorts (HC, PD, and SWEDD) reported significantly higher anxious mood, similar to other studies [30–34]; worse constipation, also described before [11, 13, 14]; and more thermoregulatory dysfunction. Thermoregulation is common in Parkinson's disease [35] and under continuous control of the autonomic, endocrine, and behavioral systems. Dysregulation in PD involves intolerance to heat and cold, hyperhidrosis, and nocturnal sweating which greatly affects the quality of life. Both sexes experienced advancing thermoregulatory dysfunction, but the mean scores were significantly higher in females. At onset, males experienced more hyposmia, and over 7 years of progression—significantly higher cognitive decline, sleep, sexual, and urinary dysfunction. Mild cognitive impairment is one of the most common NMS in Parkinson's disease [15, 16, 36]. It is established that females with PD retain a better sense of smell compared to males [37–39] and our data corroborated these findings. Males experienced a weaker stream of urine, similar to a report in fifty Parkinson's patients [17], but conflicting with two other reports [12, 18]. More than half of all PD subjects experience poor quality of sleep [40]. Understanding the pathogenesis of underlying sleep disturbances in PD is critical because it significantly impacts the quality of life. Sleep and circadian rhythm disorders disrupt the autonomic system balance [26–29] and contribute to the variety of nonmotor symptoms in Parkinson's disease.

In terms of laterality, subjects with right-sided motor predominance experienced significant sexual and urinary dysfunction. However, these results may be skewed towards males which comprised 64.2% of the sample. PD males experience difficulty with erectile dysfunction, problems with ejaculation, loss of lubrication, and sometimes hypersexuality [41]. PD patients are known to develop more pelvic organ dysfunction (bowel, bladder, and genitals) which greatly affects their quality of life [42]. Additionally, they experience involuntary micturition, weak stream of urine, storage, and voiding urinary difficulties [43]. Bladder dysfunction in PD can be linked to loss of dopaminergic neurons that project to the caudate nucleus [43]. As neural inputs are lost during disease progression, the pontine micturition center is released from tonic inhibition, which results in increased detrusor muscle activity and decreased bladder capacity [44].

Subjects with left-sided motor predominance experienced significantly more anxious mood at the onset of PD which continued as the disease progressed. It is well established that PD subjects experience more anxiety than healthy controls, with generalized anxiety disorder being the most common diagnosis [45, 46]. Interestingly, anxiety has been associated with left hemisphere involvement, which could be a manifestation of disruption in functional connectivity between the sensorimotor cortex and nigrostriatal pathway.

We were particularly interested in examining the influence of laterality on constipation and hyposmia as it has been proposed that pathologic alpha-synuclein is first detected in the enteric plexuses and olfactory pathways and later spread to involve specific regions of the brain [47]. These two symptoms often precede motor symptoms of PD by years to decades and are strong makers of prodromal PD [48]. Based on our analyses, female PD subjects reported higher mean values for constipation at onset and during the first four years of Parkinson's progression. By laterality, left-sided motor predominant subjects experienced more constipation at later time points. There were no differences in hyposmia between right and left-sided motor predominant subjects at onset. Unfortunately, hyposmia was not assessed again after the onset visit; therefore, we were not able to determine the effects of laterality or sex on its progression.

### 4.2. SWEDD Cohort

The SWEDD cohort was relatively small (*n* = 81) and we only assessed onset data for NMS for this group. Some of the PPMI participants met the clinical diagnostic criteria for PD but also had SPECT scans that did not show evidence of dopaminergic deficit (SWEDD). SWEDD subjects encompass a heterogenous group of clinical phenotypes and some of these patients may have been misdiagnosed for essential tremor, dystonia, fragile *X* premutation, iatrogenic/tardive, vascular or brain neoplasms, psychogenic, supranigral parkinsonism, and soft extrapyramidal signs of the elderly, thus at least partially explaining why this cohort experienced a high degree of NMS burden [49–51]. We were interested in this group manifesting Parkinson-like symptoms without the dopamine deficit, hoping common NMS patterns would distinguish this cohort and even point towards unknown etiologies. We found that SWEDD subjects experienced significantly more nonmotor symptoms than PD subjects. The most unique NMS that distinguished SWEDD subjects from Parkinson's subjects were the absence of hyposmia in the SWEDD group measured by the UPSIT scale, where mean values were similar to the healthy controls. SWEDD subjects with right-sided motor predominance had a higher prevalence of cardiovascular dysfunction, thermoregulatory dysfunction, cognitive decline, and impulse control dysfunction. Unfortunately, there was not sufficient data available to analyze the progression of NMS in the SWEDD cohort at the time of analyses (only 2 visits i.e., 24 months). As the SWEDD cohort of the PPMI study ages, future data should clarify differences in the progression of nonmotor symptoms.

Strength and limitations: there were several strengths to our study. We analyzed a large cohort of subjects (241 HC, 489 PD, 81 SWEDD) at onset as well as the progression of NMS for up to 7 years in PD. We examined both well-established scales of NMS–MDS UPRDS-I and SCOPA-AUT as well as subscore scales. We tested the influence of sex and laterality on NMS for a large cohort. One of the limitations of our research was the inability to study NMS on its own, without the effects of Parkinson's drug treatment. Levodopa and dopamine agonists may play a role in the progression of NMS. The SWEDD sample size was small (*n* = 81). For progression analyses, the later time points (∼6 and 7 years) had relatively smaller sample sizes due to attrition rates that are typical of longitudinal studies.

## 5. Conclusions

We identified that sex of the patient had a strong influence on the progression of NMS in PD. Males reported significantly more sexual, urinary, sleep, and cognitive dysfunction as Parkinson's progressed. Left-sided motor predominant PD patients experienced significantly more anxious moods. These data are valuable to improve PD patients' quality of life by therapeutic interventions targeting predictable NMS. Additionally, PD is a heterogenous disease comprising of motor and nonmotor symptomatology. Real time experiments investigating the role of laterality and sex on nonmotor symptoms should be pursued vehemently in animal models following asymmetrical exposures to Parkinson-inducing agents, such as chemicals, bacterial amyloid, and preformed alpha-synuclein fibrils [52–55]. Examining the onset and temporospatial spread of NMS and motor symptoms may provide clues to halting PD.

## Figures and Tables

**Table 1:** Subject characteristics.

	Healthy controls (*n* = 241)	Parkinson's disease (*n* = 489)	SWEDD (*n* = 81)
Age at study entry (mean ± SD)	60.3 ± 11.2	61.1 ± 9.7	60.4 ± 9.9
Age at disease onset (mean ± SD)	—	59.7 ± 10.0	58.7 ± 10.4

*Gender*	*n* (%)		
Female	84 (34.9)	175 (35.8)	35.8 (29)
Male	157 (65.1)	314 (64.2)	64.2 (52)

*Ethnicity*	n (%)		
Caucasian	226 (90.8)	459 (90.9)	78 (92.9)
African American	12 (4.8)	7 (1.4)	1 (1.2)
Hispanic	7 (2.8)	10 (2.0)	2 (2.4)
Asian	1 (0.4)	14 (2.8)	1 (1.2)
Indian/Alaska native	1 (0.4)	4 (0.8)	1 (1.2)
Not specified	2 (0.8)	11 (2.2)	1 (1.2)

*Handedness*	*n* (%)		
Left	24 (12)	40 (9.3)	10 (15.2)
Right	165 (82.5)	380 (88.2)	53 (80.3)
Ambidextrous	11 (5.5)	11 (2.6)	3 (4.5)

*Laterality of motor predominance*	*n* (%)		
*Female*			
** **Left	—	75 (50.3)	4 (16.7)
** **Right	—	73 (49.0)	19 (79.2)
** **Bilateral	—	1 (0.7)	1 (4.2)
*Male*			
** **Left	—	107 (37.9)	12 (28.6)
** **Right	—	165 (58.5)	26 (61.9)
** **Bilateral	—	10 (3.5)	4 (9.5)

*Hoehn and Yahr stage*	*n* (%)		
Stage 0	196 (99.0)	0	0
Stage I	2 (1.0)	187 (43.5)	38 (57.6)
Stage II	0	241 (56.0)	27 (40.9)
Stage III	0	2 (0.5)	1 (1.5)

Demographic data for all subjects. SWEDD = scans without evidence of dopaminergic degeneration.

## Data Availability

PPMI is an observational clinical study to verify progression markers in Parkinson's disease. The up-to-date information on the study and clinical data utilized for this manuscript are easily accessible in real time through this website http://www.ppmi-info.org. Data used in the preparation of this article were obtained from the Parkinson's Progression Markers Initiative (PPMI) database (http://www.ppmi-info.org/data).
